# Cross-Cultural Measurement Invariance of Scales Assessing Stigma and Attitude to Seeking Professional Psychological Help

**DOI:** 10.3389/fpsyg.2019.01249

**Published:** 2019-05-31

**Authors:** Yan Zhou, Gunnar Lemmer, Jing Xu, Winfried Rief

**Affiliations:** ^1^Department of Psychology, Philipps University of Marburg, Marburg, Germany; ^2^Department of Marxism, Zhengzhou University, Zhengzhou, China

**Keywords:** cross-culture, measurement invariance, stigma, attitudes, psychological help

## Abstract

There has been a growing interest in research on stigma and attitude toward psychotherapy, and these variables are expected to show cross-cultural variations. The Stigma Scale for Receiving Psychological Help (SSRPH), the Self-Stigma of Seeking Help (SSOSH) and the Inventory of Attitudes to Seeking Mental Health Services (IASMHS) are widely used and this study examined their measurement invariance as this is a prerequisite for use in cross-cultural studies. Data were collected online from groups of Chinese students in China (*n* = 413) and German students in Germany (*n* = 416). Confirmatory factor analyses in single samples and measurement invariance testing in a multi-group framework were conducted to test the cross-group equivalence. Findings demonstrate that the SSRPH and the modified model of IASMHS had partial scalar measurement invariance, but the SSOSH showed cultural variance in factor structure. Comparisons of latent means indicated no differences between the two groups with respect to the social stigma attached to professional psychological help, but a higher psychological openness of Chinese students toward help-seeking. Findings are discussed from intercultural and methodological perspectives. In the future, intercultural cooperation should be promoted in order to develop a cross-culturally valid concept of stigma against psychological help that could be used as the basis for intercultural comparison and developing interventions to reduce stigma.

## Introduction

Psychological interventions are now used as first-line treatments for many mental disorders, but access to and take up of psychotherapy strongly depend on stigma and attitudes to these treatments. Studies have shown that Asians, including Chinese, have more negative attitudes to help-seeking (Parker et al., 2006; Chen and Mak, 2008; Jimenez et al., 2012) and use mental health services less frequently than Westerners (Kim, 2007; Leong et al., 2007; Wang et al., 2007). Most of this research was based on samples of Chinese people living in Western countries (Parker et al., 2006; Mellor et al., 2013; Papadopoulos et al., 2013; Yang et al., 2013); only a few studies have directly investigated how attitude to psychotherapy differs between mainland Chinese and Westerners (Chen and Mak, 2008). Although modern psychotherapy has a shorter history in China than in Western countries such as Germany, it has been rapidly expanding for the past 30 years (Zhao, 2017). Since China adopted policies of reform and greater openness in 1978, not only has it undergone rapid economic development, urban Chinese have been influenced by Western science, lifestyle, and individualism (Kolstad and Gjesvik, 2014). It has been assumed that traditional and Western values coexist in urban areas in China such that urban and educated Chinese are now likely to have similar perceptions of mental illnesses to Westerners and cope with them in a similar way (Kolstad and Gjesvik, 2014). On the other hand, the perception of the stigma toward mental illness and the public’s willingness to seeking professional psychological help have improved in western countries such as Germany over the last two decades (Angermeyer et al., 2013, 2014). However, stigma and negative attitudes toward psychotherapy are still widespread. In a study conducted by Albani et al. (2013), about a third of the respondents could not even imagine having to undergo psychotherapy. Furthermore, 7.9% expressed they would prefer avoiding any contact with a neighbor if he told them that he is currently undergoing psychotherapy (Albani et al., 2013). However, an in-depth cross-cultural examination of the differences pertaining to the stigma and attitudes toward professional psychological help is overdue.

Investigating potential differences on these measures is indicated, because the role of psychological interventions between Chinese and German cultures is very different. While psychotherapy is established as a typical treatment in Germany for 100 years, its history in China is much shorter, and influenced by other developments of the Chinese health care system and Chinese traditions. So, the first step in intercultural comparisons is to examine whether the assessment tools are cross-culturally valid (Miller and Sheu, 2008). Scales are considered comparable across cultures when the measurement relationship between the observed indicators and their underlying latent variables is the same in different cultural groups (Vandenberg and Lance, 2000). Country differences in scale means can result from differences in understanding of certain concepts, translation problems or other measurement errors and may mean that a scale fails to capture interesting substantial differences accurately.

There are two approaches of measurement equivalence analysis: differential item functioning (DIF) and confirmatory factory analysis (CFA). Although these two approaches have the similar concepts and procedures (Meade and Lautenschlager, 2004; Stark et al., 2006), we preferred the CFA because that DIF analysis for multidimensional models are less established and it is more appropriate for multiple choice test data (Tay et al., 2015). CFA offers a robust statistical framework for testing measurement equivalence, also called measurement invariance.

Miller and Sheu (2008) suggested three most frequently assessed levels of measurement invariance: configural, metric, and scalar invariance. The aforementioned levels of invariance are inherent in a hierarchical structure; thus, they build on one another. Configural invariance is present when the number of factors and the pattern of the factor loadings between the latent variables and indicators in the compared groups are similar. Subsequently, metric invariance is present when the factor loadings of items are invariant across groups. The strict factorial invariance/scalar invariance is present when both the factor loadings and intercepts are invariant across groups. In terms of content, this level of invariance signifies that the observed feature can be mapped across the groups on a common scale, for instance, by having a common zero point. Evidence for scalar invariance or at least partial scalar invariance^1^ is a prerequisite for the comparison of latent mean values obtained from sub-samples (Bryne et al., 1989; Brown, 2006). Non-invariance can provide important information about the way different groups interpret the same construct (Putnick and Bornstein, 2016).

Measurement equivalence should be examined before a scale is used for cross-culture comparisons. Nevertheless, few studies (Vogel et al., 2013a) have examined the cross-cultural measurement invariance of questionnaires measuring stigma and attitudes to psychotherapy. To address this limitation, we examined the cross-cultural validity of stigma and attitude to psychotherapy scales in China and Germany.

Corrigan and Watson (2002) distinguished two types of stigma, self-stigma, and public stigma. We opted to assess the scales most commonly used to measure these types of stigma in empirical research.

Public stigma is defined as a set of negative attitudes and beliefs that motivate fear, rejection, avoidance, and discrimination against people with mental illness (Corrigan and Watson, 2002). A longitudinal study (Vogel et al., 2013b) found that public stigma predicts self-stigma (defined in the next paragraph) 3 months later. Public stigma is mostly assessed using the one-dimensional Stigma Scale for Receiving Psychological Help (SSRPH; Komiya et al., 2000).

Self-stigma consists of the application of stereotypes and prejudices against people with mental illness to oneself and the resulting self-discrimination (Corrigan and Watson, 2002). It results in low-esteem, low self-efficacy, and failure to take advantage of health care opportunities (Corrigan and Watson, 2002). The hypothesis that self-stigma is associated with a negative attitude to seeking help for mental illness has been confirmed in diverse samples including students (Cheng et al., 2015, 2018; Jennings et al., 2015; McDermott et al., 2017; Mullen and Crowe, 2017). The Self-Stigma of Seeking Help Scale (SSOSH) assesses perceptions of the loss of self-esteem the respondent would feel if he or she decided to seek professional psychological help (Vogel et al., 2006). A brief report by Vogel et al. (2013a) provided evidence of the measurement equivalence of the SSOSH across groups collected from Taiwan and areas in England, Greece, Israel, Turkey, and the United States.

Attitude toward seeking professional help for mental illnesses is related to interpersonal processes and personality components involved in general help-seeking when affected by mental illnesses (Fischer and Turner, 1970). People may be deterred from seeking help for a mental health problem by fear of being stigmatized, unwillingness or inability to disclose feelings and experiences, personal preconceptions and beliefs about professional treatment (Fischer and Turner, 1970). Attitude to seeking professional help is often assessed using the 24-item Inventory of Attitudes to Seeking Mental Health Services (IASMHS; Mackenzie et al., 2004), a revised short version of Fischer and Turner’s (1970) Attitudes to Seeking Professional Psychological Help scale (ATSPPH). The IASMHS examines three aspects of attitude to help-seeking: psychological openness, help-seeking propensity, and indifference to stigma. Fang et al. (2011) showed that the help-seeking construct as operationalized by the short form of the ATSPPH (ATSPPH-SF) may not be valid for the Chinese population, because CFA failed to confirm that Chinese response data fitted the original one-factor model of the English-language ATSPPH-SF. Despite this finding, the psychometric properties of the original three-factor model of the Chinese version of the IASMHS and the cross-cultural measurement invariance of the IASMHS have still not been assessed.

This study was conducted to investigate the measurement invariance of the SSRPH, SSOSH, and IASMHS between groups of German students in Germany and Chinese students in China. Investigating students has the advantage of offering comparability in educational status and age combined with differences in cultural background. Previous studies report evidence of measurement equivalence between western and non-western countries for self-stigma assessed with SSOSH (Vogel et al., 2013a), and problems replicating the factor structure of the Attitudes to Seeking Professional Psychological Help scale (ATSPPH, Fischer and Turner, 1970) which is related to IASMHS (Fang et al., 2011). Therefore, we expected that measurement invariance of the SSRPH and the SSOSH between the two cultures is given, but the measurement invariance of the IASMHS may be problematical. We also assessed differences in latent means for public stigma, self-stigma, and attitude to seeking professional mental help where scalar measurement invariance across groups was demonstrated. Investigating the cross-cultural equivalence of the SSRPH, SSOSH, and IASMHS is a prerequisite for cross-cultural comparisons and thus enhances the validity of conclusions from such research. Testing the measurement equivalence of the three scales in Mainland China and Germany was so far not conducted.

## Materials and Methods

### Participants

Data collection in Germany and China started in August of 2016 and lasted 6 months. German students of the University of Marburg (total number of students: 26,355) were contacted via the university email list, and they were incentivised to participate by means of the promise that the participants would be entered in a draw for vouchers worth 20 Euros. Eventually, 456 German students took part in this study. After applying the exclusion criteria (without a migration background and confirming a minimum processing time on the scales of 10 min), the number of German students whose data was viable for analysis decreased to 416. Since the students from the University of Zhengzhou do not communicate via university email, the students’ sample in China was recruited on WeChat, a popular social media platform used by most Chinese students. The questionnaire was posted in the WeChat groups of various affiliated faculties (e.g., Economics and Electronic Information Engineering). Moreover, the students in these groups were invited to send the link to the questionnaire to students from other faculties. In total, 9,156 students were invited to participate in this study, 566 of whom participated in it. According to the same exclusion criteria as in the German sample, the number of Chinese students whose data were usable for analysis decreased to 413. These participants did not receive any financial reward. The demographic characteristics of the two groups are summarized in Table 1.^2^ The study was approved by the Ethics Committee of the Faculty of Psychology of the University of Marburg.

**Table 1:** Demographic characteristics of participants.

Variables	German students (*n* = 416)	Chinese students (*n* = 413)	*p*-value	Effect size
Sex: female *n* (%)	294 (70.7%)	250 (60.5%)	0.001	φ = 0.15
Age in years (*M* ±*SD*; range)	23.85 ± 4.66; 18–58	20.79 ± 2.57; 17–39	*p* < 0.001	Cohen’s *d* = 0.81
Highest academic degree			*p* < 0.001	Cramér’s *V* = 0.78
Bachelor (%)	183 (44%)	391 (94.7%)		
Master (%)	83 (20%)	7 (1.7%)		
Ph.D. (%)	17 (4.1%)	4 (1.0%)		
Other (%)	137 (32%)	11 (2.7%)		

*The comparisons of sex and academic degree were calculated using Chi-squared test. The comparison of the age was calculated using T-test*.

### Assessment Instruments

#### Stigma Scale for Receiving Psychological Help (SSRPH)

The social stigma of receiving psychological help was assessed with the 5-item SSRPH (Komiya et al., 2000). Participants responded to statements such as ‘Seeing a psychologist for emotional or interpersonal problems carries social stigma’ using a 4-point Likert-scale ranging from 0 (*strongly disagree*) to 3 (*strongly agree*). Total score could range from 0 to 15. The English version of SSRPH showed an acceptable level of internal consistency (Cronbach’s alpha = 0.72) and construct validity and had a one-factor structure in a sample of college students (Komiya et al., 2000). Similar internal consistency (Cronbach’s alpha = 0.71) was reported in a sample of Asian American students (Miville and Constantine, 2007). Pinto et al. (2015) examined the construct validity of the SSRPH with a sample of adolescent girls in the United States and found that the confirmatory factor analysis revealed excellent model fit for a one factor model. No previous study has examined the cross-cultural measurement invariance of the SSRPH.

#### Self-Stigma of Seeking Help (SSOSH)

Self-stigma of receiving psychological help was assessed with the SSOSH (Vogel et al., 2006), which consists of 10 items, such as ‘I would feel inadequate if I went to a therapist for psychological help,’ and ‘I would feel worse about myself if I could not solve my own problems.’ Items were rated from 1 (*strongly disagree*) to 5 (*strongly agree*). Total score could range from 10 to 50. In previous research by Vogel et al. (2006) in a sample of college students in the United States, the SSOSH had good reliability (α = 0.91) and construct validity and a one-factor structure. Vogel et al. (2006) also reported criterion validity and predictive validity of SSOSH. SSOSH score can predict attitude to professional help and intention to seek professional psychological help (Vogel et al., 2006). In a German general population sample, the SSOSH demonstrated good reliability (Cronbach’s alpha = 0.84), (Apolinário-Hagen et al., 2016). In middle school and high school children from Beijing, China, the SSOSH showed an internal consistency of *a* = 0.75 (Chen et al., 2014). Measurement invariance of the SSOSH across cultures has barely been studied. Vogel et al. (2013a) ascertained that the one-factor model of SSOSH showed measurement invariance across samples collected from six areas of the world including the United States and Taiwan.

#### Inventory of Attitudes to Seeking Mental Health Services (IASMHS)

Attitude to seeking professional psychological service was assessed using the 24-item IASMHS (Mackenzie et al., 2004), which consists of three internally consistent factors (Cronbach’s alpha = 0.87), each measured with eight items: psychological openness (Cronbach’s alpha = 0.82; sample item: ‘There are certain problems which should not be discussed outside of one’s immediate family’), help-seeking propensity (Cronbach’s alpha = 0.76; sample item: ‘If I believed I was having a mental breakdown, my first inclination would be to get professional attention.’) and indifference to stigma (Cronbach’s alpha = 0.79; sample item: ‘I would feel uneasy going to a professional because of what some people would think.’). The internal consistency of the full-scale IASMHS was 0.87. Psychological openness is an openness to acknowledging psychological problems and the possibility of seeking professional help for them. Help-seeking propensity reflects the extent to which individuals consider themselves willing and able to seek professional psychological help. Indifference to stigma captures concern about what important others would think if they were to find out that one was seeking professional psychological help. Participants responded on a five-point Likert scale ranging from 0 (*disagree*) to 4 (*agree*). Sum scores for the subscales can range from 0 to 32. The English IASMHS had high reliability and validity in previous research (Mackenzie et al., 2004). The Chinese version demonstrated satisfactory reliability (Cronbach’s alpha = 0.77) in a sample of Macao citizens (Found, 2016) and similar reliability in a sample of college students in Taiwan (Loo et al., 2011). No previous study has examined the cross-cultural measurement invariance of the IASMHS. Fang et al. (2011) investigated the psychometric properties of the ATSPPH-SF, a short form of the IASMHS, with a sample of college students in Mainland China. By performing confirmatory factor analysis, the researcher determined that the help-seeking construct, as operationalized by the ATSPPH-SF, might not be valid for Chinese students.

### Translation

The German version of IASMHS was translated and validated by Kessler et al. (2015) from the original English-language scale (Mackenzie et al., 2004). German and Chinese versions of SSRPH, SSOSH, and a Chinese version of the IASMHS were constructed using the customary translation-back-translation method recommended by Brislin (1970). The English version of the scales was initially translated to Chinese and German. Afterwards, the Chinese and German versions were translated back into English. The arising Semantic differences were discussed, and the final versions of the translation were agreed upon. The translators were native Chinese and German speakers who had excellent English reading and writing skills. The translated Chinese and German versions we used for the current study have been included in the part of Supplementary Tables S5, S6.

### Statistical Analyses

Only data from students who had been born and raised in the country concerned were included in the groups of Chinese students in China and German students in Germany. Measurement invariance analysis was conducted according to the procedure described by Vandenberg and Lance (2000), van de Schoot et al. (2012), and used by Schulte et al. (2013), which provided multi-group comparisons in the context of CFA. CFAs were conducted with the software program M*plus* v7.4 (Muthén and Muthén, 2015) because of its flexibility (van de Schoot et al., 2012).

First, we examined separate measurement models for each group using confirmatory factor analyses (CFA). Decisions about goodness of model fit were based on χ^2^ difference tests, as recommended by Hu and Bentler (1999). Because the χ^2^ difference test is sensitive to sample size, we also used several other common indices to evaluate goodness of fit (Weiber and Mühlhaus, 2015: χ^2^/*df* ≤ 3, CFI ≥ 0.95, RMSEA ≤ 0.08, SRMR ≤ 0.08; Ford et al., 1986: factor loadings of items should be greater than 0.40). In case of model misspecifications, item difficulty and item discrimination were also examined (item difficulty should be between 20 and 80; Lienert and Raatz, 1998: item discrimination should be more than 0.30). Examination of measurement invariance was be repeated by excluding items with sub-optimal item discrimination and item difficulty.

Second, the step-up approach was used to add a series of increasingly stringent equality constraints to the models (Vandenberg and Lance, 2000; Brown, 2006). Multiple group comparisons were used to test the configural invariance of the baseline model. No equality constraints were imposed at this stage. We investigated whether the number of the factor and general loading pattern were the same across groups. Next, factor loadings of indicators were constrained to be equal across groups in order to examine metric invariance. To examine the next highest form of measurement invariance, scalar invariance, intercepts of indicators were also constrained to be equal across groups. Gradual equality constraining of the parameters across groups will lead to a decrease in terms of the model fit. In respective of whether a model is accepted or rejected, the decision was based on the χ^2^ difference tests (Hu and Bentler, 1999). Since the χ^2^ difference tests were sensitive to the sample size, we have additionally determined that the difference in CFI between the base model and the constrained model should not be more than 0.01 (Cheung and Rensvold, 2002). Furthermore, partial invariance was examined in case full measurement invariance could not be established (Bryne et al., 1989). By means of modification indices, a modified model for checking partial invariance by releasing the equality constraints in the descending order for misspecified items, was subsequently examined. To establish partial measurement invariance, at least two loadings or intercepts should be equal across groups (Bryne et al., 1989).

Third, latent means were compared if there was evidence of scalar invariance or partial scalar invariance. The latent mean of one group was fixed to zero, and the latent mean of the other group was allowed to freely estimate. Then the differences between the latent means was examined.

## Results

### Descriptive Statistics

Table 2 shows the internal consistencies, means, standard deviations, skewness and kurtosis of the sum scores for each scale in each group. Based on Kline’s (2010) criteria (skewness ≤ 3, kurtosis ≤ 8) we concluded that the data were normally distributed. In the sample of German students, the internal consistency was good (α > 0.70) for all scales. In the Chinese group, SSRPH (α = 0.67) and the psychological openness subscale of the IASMHS (α = 0.62) showed less than good internal consistency. Item 5 in the German version of the SSOSH, ‘My view of myself would not change just because I made the choice to see a therapist,’ showed an unexpectedly small correlation (<0.10) with other items of the scale. Items 6 and 18 of the IASMHS demonstrated unexpectedly small correlations (<0.10) with most of the other items of the scale in the German group. In the group of Chinese students, all IASMHS items showed small correlations (<0.10) with at least five other items. Despite these findings, we used CFA to test the original models in both groups. In order to examine the characteristic of the items in more detail, we also checked item difficulty and item discrimination of the three scales (Supplementary Tables S1, S2).

**Table 2:** Means, standard deviations, skewness, kurtosis, and internal consistency across scales and groups.

	German students					Chinese students				
Scale	*M*	*SD*	Skew	Kurt	*α*	*M*	*SD*	Skew	Kurt	*α*
SSOSH	11.65	4.41	0.30	−0.48	0.80	7.86	3.01	1.12	0.78	0.82
SSRPH	25.19	7.35	0.27	−0.70	0.81	24.94	4.93	−0.35	0.00	0.67
IASMHS-I	18.83	5.45	0.41	−0.17	0.70	25.55	4.12	−0.81	2.20	0.62
IASMHS-II	18.59	6.01	0.46	0.12	0.81	21.72	3.77	0.89	3.67	0.70
IASMHS-III	18.08	6.75	0.43	−0.49	0.85	18.84	4.77	0.04	−0.18	0.80

*SSRPH, Stigma Scale for Receiving Psychological Help; SSOSH, Self-stigma of Seeking Help; IASMHS-I, Psychological Openness; IASMHS-II, Help-Seeking Propensity; IASMHS - III, Indifference to Stigma; Skew, Skewness; Kurt, Kurtosis.*

### Examining the Measurement Invariance of the SSRPH

#### Examination of the Measurement Model

We started by using CFA to examine the fit of a one-factor model of the SSRPH in the two groups separately (Table 3). The unconstrained factor loadings and intercepts were presented in Supplementary Table S3. All the loadings were above 0.40. The one-factor of the SSRPH had a good fit (CFI, SRMR, and RMSEA) in both groups, except in the case of the RMSEA in data from Chinese students (RMSEA = 0.086; 90% CI [0.050-0.127]). Modification indices showed that the model fit in this group could be improved by allowing correlation of the error terms for items 1 and 5. To promote comparability across groups, the correlated error terms were not allowed in the model testing measurement invariance.

**Table 3:** Summary of fit indices from comparative factor analysis (CFA) and invariance analyses between groups for the SSRPH.

Model-SSRPH	χ^2^ (df)	CFI	RMSEA [90% CI]	SRMR	ΔCFI	Δχ^2^ (df)
*Single group CFA - original one factor model*						
German students	17.726 (5)	0.982	0.078 [0.041, 0.119]	0.026		
Chinese students	20.445 (5)	0.964	0.086 [0.050, 0.127]	0.029		
*Multiple group CFA models*						
Model A: Configural invariance	38.171 (10)	0.975	0.082 [0.056, 0.111]	0.028		
Model B: Metric invariance	53.112 (15)	0.967	0.078 [0.056, 0.102]	0.061	0.008	14.941 (5)
Model C: Scalar invariance	274.232 (19)	0.777	0.180 [0.161, 0.199]	0.148	0.190	221.12 (4)
τ_1_ free	119.367 (18)	0.911	0.117 [0.097, 0.137]	0.085	0.056	66.255 (3)
τ_1_, τ_3_ free	69.321 (17)	0.954	0.086 [0.066, 0.108]	0.066	0.013	16.209 (2)
τ_1_, τ_3_, τ_5_ free	53.243 (16)	0.967	0.075 [0.053, 0.098]	0.062	0.000	0.131 (1)

*SSRPH, Social-Stigma Scale for Receiving Psychological Help; τ_1_, ‘Seeing a psychologist for emotional or interpersonal problems carries social stigma’; τ_3_, ‘People will see a person in a less favorable way if they come to know that he/she has seen a psychologist’; τ_5_, ‘People tend to like those who are receiving professional psychological help less’. All χ^2^ tests and Δχ^2^ were significant, p < 0.001.*

#### Measurement Invariance Between Cultures

The results of multi-group tests of measurement invariance of the SSRPH are presented in Table 3. Fit indices of the baseline model of the SSRPH were in line with configural measurement invariance (χ^2^ = 38.171, *df* = 10, *p* < 0.001; CFI = 0.975; RMSEA = 0.082, 90% CI [0.056–0.111]; SRMR = 0.028). In the model imposing metric measurement invariance, item loadings were constrained to be equal between groups. A comparison of this model with the baseline model using an χ^2^ difference test showed that fit of the more restrictive model with equal factor loadings is worse than the fit of the baseline model (Δχ^2^ = 14.941, df = 5, *p* < 0.001). Because the χ^2^ difference test is sensitive to sample size, and the other common indices (CFI = 0.967; RMSEA = 0.078, 90% CI [0.056–0.102]); SRMR = 0.061; ΔCFI = 0.008) were good, we could assume that the fit of metric invariance was acceptable. To examine scalar invariance, item loadings were constrained to be equal between groups. The fit of the model testing scalar measurement invariance was poor (χ^2^ = 274.232, *df* = 19, *p* < 0.001; CFI = 0.777; RMSEA = 0.180, 90% CI [0.161–0.199]; SRMR = 0.148), and it provided a worse fit than the partial metric measurement invariance model (ΔCFI = 0.190). Modification indices showed that some indicators intercepts were not invariant across groups. Partial scalar measurement invariance was established by allowing the intercepts of items 1, 3, and 5 to vary in the descending order (χ^2^ = 53.243, *df* = 16, *p* < 0.001; CFI = 0.967; RMSEA = 0.075, 90% CI [0.053–0.098]; SRMR = 0.062; ΔCFI = 0.000).

#### Latent Mean Comparisons

Because the SSRPH showed partial scalar invariance, group comparison of latent means was possible. In multigroup comparisons of latent mean differences, the group of German students was used as the reference group with a latent mean fixed to zero. Chinese students had lower SSRPH scores (mean difference = 0.129) than German students, indicating that the Chinese students attached a fewer social stigma to seeking professional psychological help, but the mean difference was not significant (*p* > 0.05).

### Examining the Measurement Invariance of the SSOSH

The corresponding CFA showed that the measurement model of the SSOSH had a good fit (Table 4) only for German students (χ^2^ = 76.407, *df* = 27, *p* < 0.001; CFI = 0.963; RMSEA = 0.066, 90% CI [0.049, 0.084]; SRMR = 0.034; Chinese students: χ^2^ = 316.601, *df* = 27, *p* < 0.001; CFI = 0.660; RMSEA = 0.161, 90% CI [0.145–0.177]; SRMR = 0.121). To improve the baseline model for the Chinese group, both item discrimination and item difficulty were examined. To elaborate, item 2 (“My self-confidence would NOT be threatened if I sought professional help”), 4 (“My self-esteem would increase if I talked to a therapist”), 5 (“My view of myself would not change just because I made the choice to see a therapist”), and 10 (“I would feel worse about myself if I could not solve my own problems”) showed low item discrimination (<0.30, Supplementary Table S1), which indicated that the value of these items corresponded poor to the scale. Subsequently, the examination of measurement invariance was repeated by excluding these items (Table 4), and the measurement model of the SSOSH in the Chinese group was improved [Δχ^2^ (*df*) = 196.531 (18), *p* < 0.001; ΔCFI = 0.162]. However, the general model fit was still unacceptable (CFI = 0.826; RMSEA = 0.173, 90% CI [0.146–0.201]; SRMR = 0.084). This suggests that the SSOSH, which was developed to measure a Western concept, cannot measure an identical “self-stigma of seeking professional psychological help” construct in Chinese students. Hence, no baseline model for the analysis of measurement invariance between the groups could be established, and the comparison of the latent means could not be conducted.

**Table 4:** Summary of fit indices from comparative factor analysis (CFA) and invariance analyses across groups for the SSOSH.

Model-SSOSH	χ^2^ (df)	CFI	RMSEA [90% CI]	SRMR	ΔCFI	Δχ^2^ (df)
*Single group CFA - original one-factor model*						
German students	76.407 (27)	0.963	0.066 [0.049, 0.084]	0.034		
Chinese students	316.601 (27)	0.660	0.161 [0.145, 0.177]	0.121		
*Single group CFA – one-factor model without item 2, 4, 5, 10*						
German students	38.980 (9)	0.965	0.089 [0.062, 0.119]	0.031	0.002	37.427 (18), *p* < 0.005
Chinese students	120.070 (9)	0.826	0.173 [0.146, 0.201]	0.084	0.162	196.531 (18), *p* < 0.001

### Examining the Measurement Invariance of the IASMHS

#### Examination of the Measurement Model

Separate CFAs of the IASMHS (Table 5) showed that the original three-factor model had an acceptable RMSEA and SRMR in both groups (Chinese group: RMSEA = 0.063, 90% CI [0.057–0.069]; SRMR = 0.070; German group: RMSEA = 0.054, 90% CI [0.048–0.060]; SRMR = 0.054), but a poor general measurement model fit in both groups (Chinese groups: χ^2^ = 659.900, *df* = 249, *p* < 0.001; CFI = 0.781; German group: χ^2^ = 548.504, *df* = 249, *p* < 0.001; CFI = 0.898). After the examination of item discrimination and item difficulty, the total items of factor 2 (“help-seeking propensity”) of the IASMHS, the item 1, 4, 7, 12, 14, 18 of factor 1 (“psychological openness”), and the item 3 of the factor 3 (“indifference to stigma”) were excluded because of the low item discrimination (<0.30, Supplementary Table S2). The measurement invariance of a two-factor model (with 2 items of factor “psychological openness,” and 7 items of factor “indifference to stigma”) of IASMHS was then examined. The unconstrained factor loadings and intercepts were presented in Supplementary Table S4. All the loadings were above 0.40, except that the factor loading of item 23 in the Chinese sample was 0.39. The measurement model fit of both groups were obviously improved (Chinese group: CFI = 975; RMSEA = 0.045, 90% CI [0.024–0.065]; SRMR = 0.034; ΔCFI = 0.036; German group: CFI = 0.936; SRMR = 0.044; ΔCFI = 0.038), except that the RMSEA (0.087, 90% CI [0.070–0.104]) in the German group exceeded slightly 0.080. Baseline model for analysis of measurement invariance between the groups could be established.

**Table 5:** Summary of fit indices from comparative factor analysis (CFA) and invariance analyses across groups for the IASMHS.

Model-IASMHS	χ^2^ (df)	CFI	RMSEA [90% CI]	SRMR	ΔCFI	Δχ^2^ (df)
*Single group CFA - original three-factor model*						
German students	548.504 (249)	0.898	0.054 [0.048, 0.060]	0.054		
Chinese students	659.900 (249)	0.781	0.063 [0.057, 0.069]	0.070		
*Single group CFA - two-factor model: factor 1 (openness): item 9, 21; factor 2 (stigma): item 6, 11, 16, 17, 20, 23, 24*						
German students	107.109 (26)	0.936	0.087 [0.070, 0.104]	0.044	0.038	441.395 (223)
Chinese students	48.040 (26)	0.975	0.045 [0.024, 0.065]	0.034	0.036	611.860 (223)
*Multiple group CFA models*						
Model A: Configural invariance	155.149 (52)	0.952	0.069 [0.057, 0.082]	0.039		
Model B: Metric invariance	212.802 (61)	0.930	0.077 [0.066, 0.089]	0.123	0.022	57.653 (9)
λ_20_ free	197.567 (60)	0.936	0.074 [0.063, 0.086]	0.105	0.016	42.418 (8)
λ_20_, λ_23_ free	186.125 (59)	0.941	0.072 [0.061, 0.084]	0.093	0.011	30.976 (7)
λ_20_, λ_23_, λ_16_ free	178.899 (58)	0.944	0.071 [0.059, 0.083]	0.079	0.008	23.750 (3)
Model C: Scalar invariance	562.806 (65)	0.769	0.136 [0.126, 0.146]	0.129	0.175	383.907 (7)
τ_6_ free	413.514 (64)	0.838	0.115 [0.104, 0.125]	0.113	0.106	234.615 (6)
τ_6_, τ_23_ free	318.823 (63)	0.881	0.099 [0.088, 0.110]	0.098	0.063	139.924 (5)
τ_6_, τ_23_, τ_17_ free	247.902 (62)	0.914	0.085 [0.074, 0.096]	0.088	0.003	69.003 (4)
τ_6_, τ_23_, τ_17_, τ_24_ free	209.965 (61)	0.931	0.077 [0.066, 0.088]	0.083	0.013	31.066 (3)

*IASMHS, Inventory of Attitudes to Seeking Mental Health Services; item 6, ‘Having been mentally ill carries with it a burden of shame’; item 16, ‘I would be uncomfortable seeking professional help for psychological problems because people in my social or business circles might find out about it’; item 17, ‘Having been diagnosed with a mental disorder is a blot on a person’s life’; item 20, ‘I would feel uneasy going to a professional because of what some people would think’; item 23, ‘Had I received treatment for psychological problems, I would not feel that it ought to be “covered up”’; item 24, ‘I would be embarrassed if my neighbor saw me going into the office of a professional who deals with psychological problems.’ All χ^2^ tests and Δχ^2^ were significant, p < 0.001.*

#### Measurement Invariance Between Cultures

The results of multi-group tests of measurement invariance of the two-factor model of the SSRPH are presented in Table 5. The configural model showed a good general model fit (CFI = 0.952; RMSEA = 0.069, 90% CI [0.057, 0.082]), implying that the configural invariance could be confirmed. Then the factor loadings were constrained to be equal, and this leaded to a decrease in the general model fit, as ΔCFI exceeded 0.01 and SRMR exceeded 0.08. Modification indices indicated invariance in the item loadings. A modified model for checking partial metric invariance by releasing the equality constraints in descending order for items 20, 23, and 16 provided good fit (CFI = 0.944; RMSEA = 0.071, 90% CI [0.059–0.083]; SRMR = 0.079; ΔCFI = 0.008). Next, the factor intercepts were constrained to be equal, and the global scalar measurement invariance model had poor fit. Modification indices showed that the intercepts of items were invariant across the groups. Partial scalar measurement invariance was acceptable after allowing the intercepts of items 6, 23, 17, and 14 to vary in the descending order (CFI = 0.931; RMSEA = 0.077, 90% CI [0.049–0.081]), as SRMR (0.083) exceeded slightly 0.08 and ΔCFI (0.013) exceeded slightly 0.01. Partial scalar invariance of the two-factor model could be confirmed.

#### Latent Mean Comparisons

The comparison of the latent means was based on only two invariant items (item 9 and 21 of factor “psychological openness”). The group of German students was used as the reference group. The Chinese students had greater latent mean than German students on the two invariant items of IASMHS (*z* = 1.458, *p* < 0.001, *d* = 0.876), which means that Chinese students have less psychological openness toward seeking help than German students.

## Discussion

In the present study, we have examined measurement invariance of the SSRPH, the SSOSH and the IASMHS between Chinese students and German students, which is a prerequisite for their use in cross-culture comparisons. The results demonstrated that the SSRPH had the same factor structure in the two groups and showed partial scalar invariance. The original one-factor model of the SSOSH only fitted the group of German students. Therefore, we could not examine the measurement invariance of them between the two groups. A modified model of IASMHS with two factors had partial scalar measurement invariance. Cross-cultural comparison of latent means indicated that, in descriptive terms only, German students attached a greater stigma to seeking professional psychological help than the group of Chinese students attached least social stigma to seeking professional help, but the mean difference was not significant. The comparison of the latent means of IASMHS was based on only two invariant items, and it showed that Chinese students have less openness toward seeking help than German students.

### Stigma Scale for Receiving Psychological Help (SSRPH)

The one factor model of the SSRPH has an acceptable configural and metric invariance. At the level of scalar measurement invariance, loading of item 1 (‘Seeing a psychologist for emotional or interpersonal problems carries social stigma’) was not invariant across the groups. A re-examination of the wording indicated that the Chinese and German versions of this item were not semantically identical. The word ‘stigma’ was translated into Chinese as “
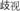
”, which emphasizes the behavioral aspects of social stigma as defined by social psychologists (Rüsch et al., 2005), whereas the German translation refers more to the cognitive aspects of social stigma toward people who seek professional psychological help. The intercepts of items 3 (“People will see a person in a less favorable way if they come to know that he/she has seen a psychologist”) and 5 (“People tend to like those who are receiving professional psychological help less”) did also not show scalar invariance across the groups. German students were more likely to agree with items 3 and 5 than the Chinese students. In Chinese culture, high power distance is highly valued (Bond, 1996). Therapists in China are viewed as experts who give directions and advice to people suffering from psychological problems (Bond, 2010). It is likely, therefore, that the Chinese students regarded people who have completed psychotherapy as having received advice from an expert that may have helped them to get their problems under control and were therefore less likely to perceive them negatively.

We identified two items (item 2: “It is a sign of personal weakness or inadequacy to see a psychologist for emotional or interpersonal problems.” and item 4: “It is advisable for a person to hide from people that he/she has seen a psychologist”) to be invariant between the two groups, which could be used for cross-cultural comparisons. Both these groups were more inclined to consider that seeking help for emotional or interpersonal problems is not a sign of personal weakness and need not be hidden from other people. This showed that in both cultures, there is a certain openness to help-seeking. While 3.28 million (33%) of 9.92 million insured persons in Germany had experiences pertaining to psychological assistance, (Gaebel et al., 2013), professional help-seeking in case of mental problems has been depicted more frequently in Chinese media (TV drama, film, and so on) in the last years, and this could have contributed to the openness among Chinese individuals regarding the same.

The differences between the latent factor means should be interpreted with caution since they are based on only two items. The SSRPH latent means of German students were higher than those of both groups of Chinese students in descriptive terms only. The SSRPH was developed in the United States using the concept of social stigma. It reflects a specific Western cultural conceptualization of social stigma and may not reflect completely the way stigma is understood in China. In China, the concept of “face” represents one’s moral capital and prestige in the social world (Kleinman and Kleinman, 1993). In Chinese society, there is a strong motivation for families to keep the existence of a family member’s mental illness a secret because extra-familial exposure of a family member’s mental illness causes loss of face of the whole family (Yang and Pearson, 2002). Further research should examine the effect of adding items that reflect concerns about negative effect for the whole family, like ‘If a family member goes to see a psychologist it brings shame on the family,’ to the SSRPH.

### Self-Stigma of Seeking Help (SSOSH)

The original one-factor model and the modified one of the SSOSH showed a poor model fit to the data of Chinese students, and no baseline model for the analysis of measurement invariance between the groups could be established. Our results did not correspond with the findings of an earlier cross-cultural study (Vogel et al., 2013a), which found that these items demonstrated partial measurement invariance across samples collected from six areas in the world including the United States and Taiwan. In this study, items 5, 9, and 10 showed variant factor loadings between the students from Taiwan and the United States. Although there are several cultural similarities between Taiwan and Mainland China, cultural differences are prevalent as well. Compared to Taiwanese college students, those from mainland China have stronger collectivist cultural inclination (Yang, 2015), and people in a collectivist society such as Mainland China are more inclined to attribute responsibility to patients for their mental disorders (Zhang and Deng, 2017). In other words, an important aspect of the self-stigma of people from Mainland China could be that they contribute mental illnesses to themselves and feel inferior because they differ from the majority of society. In this manner, they may consider that they have failed to meet social expectations and lost their social status and belonging. Thus, in future studies, researchers may take the collectivistic notions pertaining to self-stigma into account and investigate the effect of adding items such as “I would feel that I hurt the expectation of society by not solving my problems without professional help.” Due to the internalization of the prejudices related to help-seeking also in the family unit in a collectivistic society (Soheilian and Unman, 2009), Vogel et al. (2013a) suggested adding item that reflect concerns about disappointing family members (e.g., “I would feel as though I let my family down by not solving my problems without professional help”).

### Inventory of Attitudes to Seeking Mental Health Services (IASMHS)

Separate CFAs showed that the original three-factor model of the IASMHS was a poor model fit to data from the group of Chinese students. These results are similar to the findings of a study that investigated the psychometric properties of the ATSPPH-SF, a short form of the IASMHS (Fang et al., 2011). It indicated that Western concepts and models of attitudes to professional psychological are not applicable to Chinese groups. A few Chinese culture-specific aspects of the attitude toward help-seeking have not been included in the scale, such as the importance of avoid bringing shame to the family, fear of losing the “face” and the importance of emotional self-control (Kung, 2004; Kim, 2007). Furthermore, non-cultural barriers, which was found to be more salient than cultural barriers in help-seeking (Kung, 2004), should also be taken into account, for example lack of knowledge of professional psychological help, the underdevelopment of the mental health services and the financial burden of help-seeking in Chinese society. Therefore, there is a need for culturally universal instruments for assessing attitudes to seeking psychological help for the cross-cultural comparisons, but developing such instruments would not be easy, because measurement invariance is more likely to be found when culturally similar countries are compared (Rippl and Seipel, 2008), and it is probably harder to find measurement invariance in heterogeneous cultures (Schulte et al., 2013).

By examination of the measurement invariance of a modified model of IASMHS with two factors, we had found only two items (“people should work out their own problems; getting professional help should be a last resort” and “people with strong characters can get over psychological problems by themselves and would have little need for professional help”) to be invariant between the two groups and could be used for cross-cultural comparisons. It indicated that both in German and Chinese culture, it is important to solve problems through one’s own strength.

### Limitations and Conclusion

This study has some limitations. First, the sample was limited to students from China and Germany, so the results cannot be generalized to the general population of these cultures. Second, the compared samples possessed different characteristics (age, gender, and academic degree). Propensity score matching was conducted without including the variable of academic degree, since the category “other” concerning the participant’s academic degrees in the German sample referred to a specific German degree “Staatsexam,” which is equivalent to a bachelor’s degree combined with a master’s degree. Under these circumstances, it is unclear whether measurement invariance occurred due to cultural differences. For future research, it would be better to consider a variable such as “the number of semesters since bachelor” instead of the participants’ “academic degree.” Third, recruitment of a volunteer sample of students may have produced a selection bias, because those who were interested in the topic of seeking psychological help or had experience of psychotherapy may have been more likely to participate. The recruitment of the participants was carried out without a systematic selection process. Therefore, the self-selection of participants, which is a typical disadvantage of online surveys, could be detrimental to the representativeness of the random sample.

Overall, this study shows acceptable measurement invariance for the SSRPH and two items (item 9 and item 21) of IASMHS, and these scales or items could be used for cross-culture comparison. The group differences we have reported should be interpreted with caution because they are based on only the two items of each scale that demonstrated cultural invariance. The SSOSH did not show measurement invariance. Intercultural cooperation should be encouraged in order to facilitate the development of cross-cultural concepts concerning the stigma and attitude toward seeking professional psychological help, taking collectivistic notions into account, and to improve research on intercultural comparisons. This is one of the first studies to investigate the measurement invariance of the SSRPH, SSOSH, and IASMHS in large groups in China and Germany and the results should guide future research in this field. Beyond the cross-cultural comparisons, the basic concepts of stigma and attitude are crucial to improving understanding of why people reject psychological interventions even when they are recommended treatments and it has relevance for health care provision in general.

## Ethics Statement

This study was carried out in accordance with the recommendations of “Ordnung für die Lokale Ethik-Kommission des Fachbereichs Psychologie vom 10.02.2010”, die Lokale Ethik-Kommission (LEK) of University of Marburg, with written informed consent from all subjects. All subjects gave written informed consent in accordance with the Declaration of Helsinki. The protocol was approved by the ‘Ethic-Committee of Department of Psychology of University Marburg’.

## Author Contributions

YZ and WR were mainly responsible for the overall conception, design, and analysis of this study. YZ wrote the manuscript. GL made an important contribution to the revising of statistical analysis and interpretation of the data. JX contributed to sample preparation. WR and GL provided critical feedback and helped shape the manuscript.

## Conflict of Interest Statement

The authors declare that the research was conducted in the absence of any commercial or financial relationships that could be construed as a potential conflict of interest.

## Footnotes

1 Partial measurement invariance is declared when at least two items per latent variable (i.e., factor loadings, factor intercepts) are found to be invariant (Muthén and Christoffersson, 1981).

2 Since the demographic data of the two samples was significantly different, propensity score matching (by gender and age) was performed to exclude the test effect of gender and age for the purpose of data analysis. However, the results did not differ from those obtained prior to performing the propensity score matching test. The results of the comparison between the underlying structure and the invariant items of the scales remained unchanged. Therefore, we presented the results without conducting propensity score matching, owing to the large sample size.
