# Supplementary material for: Cross-Cultural Measurement Invariance of Scales Assessing Stigma and Attitude to Seeking Professional Psychological Help
Source: Front Psychol. 2019 May 31;10:1249. doi: 10.3389/fpsyg.2019.01249 (PMC6554279; doi:10.3389/fpsyg.2019.01249)
Supplement: Supplementary file 1 [file Table_1.DOCX]

***Supplementary Material***

Supplemental Table S1. Item difficulty and item discrimination of SSRPH and SSOSH

|  | Item  difficulty | | Item  discrimination | |
| --- | --- | --- | --- | --- |
|  | German sample | Chinese sample | German sample | Chinese sample |
| SSRPH |  |  |  |  |
| 1. Seeing a psychologist for emotional or interpersonal problems carries social stigma. | 44 | 8 | .56 | .59 |
| 2. It is a sign of personal weakness or inadequacy to see a psychologist for emotional or interpersonal problems. | 19 | 12 | .50 | .49 |
| 3. People will see a person in a less favorable way if they come to know that he/she has seen a psychologist. | 43 | 15 | .76. | .58 |
| 4. It is advisable for a person to hide from people that he/she has seen a psychologist. | 38 | 27 | .61 | .43 |
| 5. People tend to like less those who are receiving professional psychological help. | 23 | 10 | .62 | .60 |
| SSOSH |  |  |  |  |
| 1. I would feel inadequate if I went to a therapist for psychological help. | 41 | 33 | .66 | .42 |
| 2. My self-confidence would NOT be threatened if I sought professional help. | 45 | 42 | .36 | .25 |
| 3. Seeking psychological help would make me feel less intelligent. | 14 | 26 | .50 | .37 |
| 4. My self-esteem would increase if I talked to a therapist. | 49 | 42 | .42 | .24 |
| 5. My view of myself would not change just because I made the choice to see a therapist. | 53 | 48 | .03 | .18 |
| 6. It would make me feel inferior to ask a  therapist for help. | 35 | 20 | .70 | .50 |
| 7. I would feel okay about myself if I made the choice to seek professional help. | 26 | 39 | .70 | .46 |
| 8. If I went to a therapist, I would be less satisfied with myself. | 29 | 29 | .65 | .49 |
| 9. My self-confidence would remain the same if I sought help for a problem I could not solve. | 47 | 38 | .32 | .44 |
| 10. item dis | 42 | 58 | .67 | .06 |

Supplemental Table S2. Item difficulty and item discrimination of IASMHS

|  | Item  difficulty | | Item  discrimination | |
| --- | --- | --- | --- | --- |
|  | German sample | Chinese sample | German sample | Chinese sample |
| Factor 1: Psychological openness |  |  |  |  |
| 12. Psychological problems, like many things, tend to work out by themselves. | 21 | 49 | .42 | .17 |
| 1. There are certain problems which should not be discussed outside of one’s immediate family. | 37 | 63 | .37 | .14 |
| 21. People with strong characters can get over psychological problems by themselves and would have little need for professional help. | 27 | 49 | .49 | .31 |
| 9. People should work out their own problems; getting professional help should be a last resort. | 24 | 50 | .52 | .32 |
| 4. Keeping one’s mind on a job is a good solution for avoiding personal worries and concerns. | 37 | 65 | .36 | .11 |
| 18. There is something admirable in the attitudes of people who are willing to cope with their conflicts and fears without resorting to professional help. | 54 | 49 | .22 | .25 |
| 14. There are experiences in my life I would not discuss with anyone. | 36 | 66 | .43 | .14 |
| 7. It is probably best not to know everything about oneself. | 35 | 49 | .32 | .22 |
| Factor 2: Help-seeking propensity |  |  |  |  |
| 19. If I believed I were having a mental breakdown, my first inclination would be to get professional attention. | 46 | 57 | .50 | .21 |
| 15. I would want to get professional help if I were worried or upset for a long period of time. | 37 | 44 | .55 | .17 |
| 8. If I were experiencing a serious psychological problem at this point in my life, I would be confident that I could find relief in psychotherapy. | 25 | 36 | .59 | .17 |
| 13. It would be relatively easy for me to find the time to see a professional for psychological problems. | 48 | 50 | .35 | .19 |
| 2. I would have a very good idea of what to do and who to talk to if I decided to seek professional help for psychological problems. | 46 | 41 | .37 | .07 |
| 10. If I were to experience psychological problems, I could get professional help if I wanted to. | 24 | 46 | .44 | .21 |
| 5. If good friends asked my advice about a psychological problem, I might recommend that they see a professional. | 24 | 40 | .47 | .13 |
| 22. I would willingly confide intimate matters to an appropriate person if I thought it might help me or a member of my family. | 16 | 30 | .50 | .12 |
| Factor 3. Indifference to stigma |  |  |  |  |
| 6. Having been mentally ill carries with it a burden of shame. | 41 | 24 | .35 | .49 |
| 24. I would be embarrassed if my neighbor saw me going into the office of a professional who deals with psychological problems. | 34 | 35 | .52 | .56 |
| 11. I would be embarrassed if my neighbor saw me going into the office of a professional who deals with psychological problems. | 21 | 33 | .51 | .45 |
| 17. Having been diagnosed with a mental disorder is a blot on a person’s life. | 31 | 24 | .48 | .49 |
| 16. I would be uncomfortable seeking professional help for psychological problems because people in my social or business circles might find out about it. | 31 | 38 | .65 | .59 |
| 20. I would feel uneasy going to a professional because of what some people would think. | 27 | 40 | .66 | .46 |
| 3. I would not want my significant other (spouse, partner, etc.) to know if I were suffering from psychological problems. | 23 | 46 | .50 | .28 |
| 23. Had I received treatment for psychological problems, I would not feel that it ought to be “covered up.”. | 45 | 32 | .47 | .32 |

*Notes*. SSRPH = Social-Stigma Scale for Receiving Psychological Help; SSOSH = Self-Stigma of Seeking Help Scale; IASMHS = Inventory of Attitudes to Seeking Mental Health Services.

Supplemental Table S3. Factor loadings and factor intercepts of the 5 Items of the SSRPH across cultures

|  | Germany sample | | Chinese sample | |
| --- | --- | --- | --- | --- |
| Item | Loading | Intercepts | Factor loading | Intercept |
| 1 | .64 | 2.25 | .68 | 1.58 |
| 2 | .51 | 1.64 | .58 | 1.43 |
| 3 | .91 | 2.25 | .69 | 1.40 |
| 4 | .66 | 2.02 | .48 | 1.60 |
| 5 | .71 | 1.86 | .64 | 1.48 |

*Notes*. SSRPH = Social-Stigma Scale for Receiving Psychological Help.

Supplemental Table S4. Factor loadings and factor intercepts of the 9 Items of the IASMHS across cultures

|  | | Germany sample | | Chinese sample | |
| --- | --- | --- | --- | --- | --- |
|  | Item | Loading | Intercepts | Factor loading | Intercept |
| Factor 1 | 21 | .66 | 1.81 | .54 | 2.99 |
|  | 9 | .67 | 1.77 | .62 | 2.93 |
| Factor 2 | 6 | .51 | 1.97 | .59 | 2.39 |
|  | 24 | .72 | 1.77 | .73 | 2.59 |
|  | 11 | .61 | 1.75 | .58 | 2.47 |
|  | 17 | .57 | 1.82 | .67 | 2.31 |
|  | 16 | .84 | 1.88 | .76 | 2.63 |
|  | 20 | .85 | 1.85 | .60 | 2.74 |
|  | 23 | .54 | 2.30 | .39 | 2.72 |

*Notes.* IASMHS = Inventory of Attitudes to Seeking Mental Health Services.

Supplemental Table S5. Chinese Version of the SSRPH, SSOSH and IASMHS

| Item | SSRPH |
| --- | --- |
| 1 | 因为个人问题去看心理医生会受到社会的歧视。 |
| 2 | 因为个人问题去看心理医生体现了个人的弱点或不足。 |
| 3 | 人们会对那些去看心理医生的人产生不好的印象。 |
| 4 | 如果有人去看了心理医生，他最好向别人隐瞒此事。 |
| 5 | 接受专业心理帮助的人会不那么受人欢迎。 |
|  | SSOSH |
| 1 | 如果我要求助心理医生，我会感觉很别扭。 |
| 2 | 我的自信心不会因为我去求助心理医生而受到威胁。 |
| 3 | 寻求心理帮助会让我觉得自己不是那么聪明。 |
| 4 | 和心理医生谈话后会使我的自信心增强。 |
| 5 | 我对自己的看法不会因为我选择去看心理医生而改变。 |
| 6 | 向心理医生寻求帮助会让我感到低人一等。 |
| 7 | 去看心理医生对我来说不是什么问题。 |
| 8 | 我将会因为去看心理医生而对自己不是那么满意。 |
| 9 | 如果我因为自己不能解决的问题去找心理医生，这将不会对我的自信心造成什么影响。 |
| 10 | 如果我解决不了自己的问题，我将会感觉很糟糕。 |
|  | IASMHS |
| 1 | 有些问题不应该在直系亲属之外讨论。 |
| 2 | 如果我决定去看心理医生，我会非常清楚我该怎样做，该求助于那些人。 |
| 3 | 我不想让我亲近的人(配偶，伴侣) 知道我遭受了心理问题的困扰。 |
| 4 | 全身心投入工作是避免个人忧虑和过分担忧的一个好办法。 |
| 5 | 如果某个好朋友向我咨询有关心理方面的问题，我或许会推荐他去看心理医生。 |
| 6 | 患有心理障碍是一件丢人的事。 |
| 7 | 也许最好不把自己了解得那么透彻。 |
| 8 | 如果我正经历一个严重的心理危机，我相信自己能从心理医生那得到帮助。 |
| 9 | 每个人都应靠自己来解决自己的问题，去看心理医生应该是最后一种选择。 |
| 10 | 如果我有心理问题，只要我愿意我就能找到专业的心理帮助。 |
| 11 | 如果我生命中重要的人发现我有心理问题，他们不会像以前那样看重我。 |
| 12 | 像许多事情一样，心理问题是可以自动解决的。 |
| 13 | 我将会很容易安排出时间去看心理医生。 |
| 14 | 生活中的某些经历我不想和任何人谈起。 |
| 15 | 如果我担忧或难过了很长一段时间，我可能会去看心理医生。 |
| 16 | 由于心理问题去看心理医生会让我感到不自在，因为这可能会被我的朋友，熟人或同事发现。 |
| 17 | 被诊断为患有心理疾病是一个人一生中的一大污点。 |
| 18 | 那些靠自己来应对内心冲突和恐惧而不去看心理医生的人是值得敬佩的。 |
| 19 | 如果我认为自己患了心理疾病，我的第一反应就是去看心理医生。 |
| 20 | 我会因为别人的看法而对寻求专业心理帮助感到不自在。 |
| 21 | 一个性格坚强的人往往能依靠自己克服心理问题，而很少需要心理医生的帮助。 |
| 22 | 如果向一个合适的人倾诉我的隐私可以帮助我或我的家人，我会去这么做。 |
| 23 | 我不会认为我曾去看过心理医生是不可告人的秘密。 |
| 24 | 如果邻居发现我去看心理医生，我会感觉很难堪。 |

*Notes*. SSRPH = Social-Stigma Scale for Receiving Psychological Help; SSOSH = Self-Stigma of Seeking Help Scale; IASMHS = Inventory of Attitudes to Seeking Mental Health Services.

Supplemental Table S6. German Version of the SSRPH, SSOSH and IASMHS

| Item | SSRPH |
| --- | --- |
| 1 | Es ist sozial stigmatisiert, wegen persönlicher oder zwischenmenschlicher Probleme zu einem Psychologen zu gehen. |
| 2 | Es ist ein Zeichen von Schwäche, wenn man wegen persönlicher oder zwischenmenschlicher Probleme zu einem Psychologen geht. |
| 3 | Wenn herauskommt, dass man psychologische Hilfe aufgesucht hat, werden andere ein schlechtes Bild von einem haben. |
| 4 | Wenn man psychologische Hilfe aufgesucht hat, sollte man das besser geheim halten. |
| 5 | Wenn man psychologische Hilfe aufsucht, wird man von anderen Menschen weniger gemocht. |
|  | SSOSH |
| 1 | Es wäre mir unangenehm, wenn ich psychologische Hilfe aufsuchen würde. |
| 2 | Mein Selbstbewusstsein würde nicht darunter leiden, wenn ich psychologische Hilfe aufsuchen würde. |
| 3 | Wenn ich psychologische Hilfe aufsuchen würde, würde ich mich weniger intelligent fühlen. |
| 4 | Mein Selbstwertgefühl würde steigen, wenn ich mit einem Therapeuten sprechen würde. |
| 5 | Mein Bild von mir selbst würde sich nicht verändern, wenn ich zu einem Therapeuten gehen würde. |
| 6 | Ich würde mich schwach fühlen, wenn ich Hilfe bei einem Therapeuten suchen würde. |
| 7 | Es wäre in Ordnung für mich, wenn ich psychologische Hilfe aufsuchen würde. |
| 8 | Wenn ich zu einem Therapeuten gehen würde, wäre ich weniger zufrieden mit mir selbst. |
| 9 | Mein Selbstbewusstsein würde sich nicht verändern, wenn ich psychologische Hilfe wegen eines Problems suchen würde, das ich selbst nicht lösen kann. |
| 10 | Ich würde mich schlecht fühlen, wenn ich meine Probleme nicht selbst lösen könnte. |
|  | IASMHS |
| 1 | Es gibt bestimmte Probleme, die man nicht außerhalb des eigenen engen Familienkreises besprechen sollte. |
| 2 | Ich habe eine klare Vorstellung davon, was zu tun wäre und wen ich ansprechen könnte, wenn ich mich dazu entscheiden würde, professionelle Hilfe (d.h. durch z.B. Psychologe oder Psychiater) wegen psychischer Probleme in Anspruch zu nehmen. |
| 3 | Ich würde nicht wollen, dass meine engen Bezugspersonen (Ehepartner, Partner, etc.) davon wüssten, wenn ich unter psychischen Problemen leiden würde. |
| 4 | Sich auf seine Arbeit zu konzentrieren ist eine gute Lösung, um persönlichen Ängsten und Sorgen aus dem Weg zu gehen. |
| 5 | Wenn ein guter Freund mich bezüglich eines psychischen Problems um Rat fragen würde, würde ich ihm/ihr möglicherweise dazu raten, professionelle Hilfe zu suchen. |
| 6 | Psychisch krank gewesen zu sein ist etwas Schambesetztes. |
| 7 | Es ist wahrscheinlich besser, nicht alles über sich selbst zu wissen. |
| 8 | Ich bin zuversichtlich, dass Psychotherapie mir helfen könnte, wenn ich zum jetzigen Zeitpunkt in meinem Leben ein ernstes psychisches Problem hätte. |
| 9 | Leute sollten ihre Probleme selbst lösen; professionelle Hilfe sollte der letzte Ausweg sein. |
| 10 | Wenn ich psychische Probleme hätte, könnte ich professionelle Hilfe bekommen, wenn ich wollte. |
| 11 | Wichtige Personen in meinem Leben würden weniger von mir halten, wenn sie herausfinden würden, dass ich psychische Probleme hätte. |
| 12 | Genau wie viele andere Dinge, lösen sich psychische Probleme meist von alleine. |
|  |  |
| 13 | Ich würde ziemlich einfach Zeit dafür auffinden, professionelle Hilfe für psychische Probleme in Anspruch zu nehmen. |
| 14 | Ich habe in meinem Leben Erfahrungen gemacht, die ich mit niemandem besprechen würde. |
| 15 | Ich würde professionelle Hilfe in Anspruch nehmen wollen, wenn ich mir über eine lange Zeit hinweg Sorgen machen würde oder unglücklich wäre. |
| 16 | Ich würde mich dabei unwohl fühlen, professionelle Hilfe wegen psychischer Probleme in Anspruch zu nehmen, weil Personen in meinem sozialen Umfeld und Arbeitsumfeld das herausfinden könnten. |
| 17 | Die Diagnose einer psychischen Erkrankung ist ein Makel im Leben einer Person. |
| 18 | Es hat etwas bewundernswertes, wenn Personen bereit sind, mit ihren Konflikten und Ängsten umzugehen ohne auf professionelle Hilfe zurückzugreifen. |
| 19 | Wenn ich denken würde, ich hätte einen Nervenzusammenbruch, wäre mein erster Schritt, professionelle Hilfe zu suchen. |
| 20 | Ich würde mich nicht dabei wohl fühlen, professionelle Hilfe in Anspruch zu nehmen, aufgrund dessen, was manche Leute wohl darüber denken würden. |
| 21 | Menschen mit einem starken Charakter können alleine mit psychischen Problemen fertig werden und haben kaum einen Bedarf an professioneller Hilfe. |
| 22 | Ich wäre bereit, persönliche Dinge mit einer geeigneten Person zu besprechen, wenn ich denken würde, dass es mir oder jemandem in meiner Familie helfen könnte. |
| 23 | Wenn ich aufgrund psychischer Probleme in Behandlung gewesen wäre, hätte ich nicht das Gefühl, dass ich das verstecken müsste. |
| 24 | Es wäre mir peinlich, wenn mein Nachbar sehen würde, dass ich in eine psychotherapeutische Praxis gehen würde. |

*Notes*. SSRPH = Social-Stigma Scale for Receiving Psychological Help; SSOSH = Self-Stigma of Seeking Help Scale; IASMHS = Inventory of Attitudes to Seeking Mental Health Services.
